# Describing a programme of implementation‐effectiveness research on the organization and implementation of frontline nursing care delivery into diverse health systems

**DOI:** 10.1111/jan.16395

**Published:** 2024-08-16

**Authors:** Miriam Bender, Marjory (Micki) Williams

**Affiliations:** ^1^ Sue & Bill Gross School of Nursing, University of California, Irvine 854 Medical Sciences Quad Irvine California USA; ^2^ Research Investigator (WOC), Research Service Central Texas Veterans Health Care System Temple Texas USA

**Keywords:** care pathways, conceptual models of nursing, delivery of health care, models of care, research implementation, work organisation

## Abstract

**Aims:**

The longitudinal programme of research described in this paper seeks to generate knowledge about factors influencing the implementation of a system‐level intervention, the clinical nurse leader care model, involving nurses as leaders at the frontlines of care and the outcomes achievable with successful implementation. The research programme has the following aims, (a) to clarify clinical nurse leader practice, (b) develop and empirically validate a translational model of frontline care delivery that includes clinical nurse leader practice and (c) delineate the patterns of and critical outcomes of successful implementation of the clinical nurse leader care model.

**Design:**

This programme of research follows a knowledge‐building trajectory involving multiple study designs in both qualitative (grounded theory, case study) and quantitative (descriptive, correlational and quasi‐experimental) traditions.

**Methods:**

Multiple mixed methods within a system‐based participatory framework were used to conduct this programme of implementation–effectiveness research.

**Results:**

Findings are demonstrating how the clinical nurse leader care model, as a complex system‐level intervention, can be implemented in diverse healthcare contexts to make a difference to patient care quality and safety. Findings also contribute to implementation science, helping to better understand the dynamic interdependencies between implementation, the interventions implemented and the contexts in which they are implemented.

**Conclusion:**

Findings translate into sets of evidence‐informed implementation ‘recipes’ that health systems can match to their specific contexts and needs. This allows health systems to take on strategies that both maximize resource impact within their existing structures and support achieving intended outcomes.

**Implication:**

This programme of research is producing actionable implementation and outcome evidence about ways to organize nursing knowledge and practice into care models that can be successfully adopted within real‐world healthcare settings to achieve safer and higher quality patient care.

**What does this paper contribute to the wider global clinical community?**

- Research that informs implementation and effectiveness of the delivery of healthcare services is relevant to public and population health and the prevention of patient morbidity and mortality associated with inadequate or inappropriate care.
- Nurses are the largest health workforce component, with identified potential to improve patient safety. What remains unclear is how to specifically organize and implement nursing knowledge and practice into care delivery models that consistently achieve national quality mandates and optimize patient outcomes.
- The research described in this paper leverages sophisticated analytic methodologies to fill gaps in knowledge in a way that can be successfully translated into real‐world healthcare settings to achieve better care and better health.

## 1 INTRODUCTION

In response to the *Journal of Advanced Nursing's* special issue call for articles that can expand understanding of implementation science in nursing, this paper will describe the orientation, development and ongoing contributions of a decade‐long programme of research conducted by nursing scholars that seek to understand how best to organize and implement nursing practice into the frontline hospital healthcare context. The care delivery model of interest in this programme of research is the clinical nurse leader (CNL) care model. This model has been highlighted by the Agency for Healthcare Research and Quality (AHRQ) (2010), the Institute of Medicine (2011) and the Robert Wood Johnson Foundation (Joynt & Kimball, 2008). The CNL is an RN with master's level competencies in clinical leadership, care environment management and clinical outcomes management. The initial presentation of CNL integrated care delivery was for the CNL to utilize these competencies as a member of the frontline clinical care staff and to take the lead in developing clinical structures and processes that improve care coordination, quality and safety. While safety and quality improvement efforts are encouraged in RN care models such as primary nursing, the required clinical leadership activities may not be consistently executed because ever‐increasing patient acuity means that staff nurses are primarily directing all their efforts towards emergent patient needs.

## 2 BACKGROUND

### 2.1 History of the CNL initiative

The CNL initiative, introduced by the American Association of Colleges of Nursing (AACN) in 2005, was responsive to and emerged into a climate of concern for healthcare system outcomes, as described in the Institute of Medicine's landmark 2000 report *To Err is Human*. The initial vision was for a new nursing role equipped with specific competencies and positioned within the system to detect, assess and influence both structural and functional healthcare patterns that are associated with desired quality and safety outcomes. The CNL was introduced as an innovation with clearly defined education and certification requirements (AACN, 2007), but without any specific attention to its implementation into healthcare delivery. As with many innovations, the initial period involved diverse experimentation across a variety of contextual environments (Williams & Bender, 2015).

A particular challenge was how to strategically integrate new nursing practices into pre‐existing healthcare delivery systems and nursing cultures defined by traditional evidence‐based expectations for nursing practice, and fiscally driven realities for nurse staffing models based on task‐defined workloads. This is not a novel scenario. As Tucker and Edmondson (2003) describe it, hospitals rely on frontline care providers for care quality outcomes yet have only recently begun to understand the importance of systematic, organizational improvement to increase care quality and safety. There is evidence to suggest that a focus on improving care delivery models in general, rather than, say, focusing on specific clinical conditions (e.g. heart failure) or interventions (e.g. checklists) can increase safety and efficiency in hospital settings (Tucker et al., 2008). While this is changing, much research still focuses mostly on interventions targeting specific patient populations rather than addressing the broader challenge of changing inter‐organization and inter‐unit care delivery processes (Shortell & Singer, 2008), such as nursing care delivery.

As Yakusheva et al. (2014) make clear, this may be because of the great difficulty in capturing the temporal dynamic effects of nursing practice as well as the interdependency of contextual factors and nursing practice, which result in insufficient levels of evidence to support the ‘case’ for investment in nursing models of care. Because of this, there remains a tension between a perception of nursing as a prohibitive cost to hospital management and the known costs of adverse patient events that research has shown nursing to positively influence (Needleman, 2017). What has been needed is a body of research that can elucidate nursing's direct and indirect mechanisms of action as organized within diverse contexts of care and that can link these actions with improved care quality and safety (Kitson, Muntlin Athlin, & Conroy, 2014).

These insights were born out in the early efforts of health systems attempting to adopt CNLs in their nursing models of care. The CNL White Paper (AACN, 2007) and anecdotal evidence regarding CNL practice provided support for healthcare system early adopters, yet this proved inadequate to support effective and reliable reorganization of frontline nursing care delivery to include CNL practice. A lack of shared understandings about CNL practice and a lack of empirical evidence necessary to support clinical and fiscal leadership decisions regarding the implementation of the CNL care model motivated the programme of research described in this paper. Prior studies had demonstrated the CNL care model's feasibility to improve frontline quality and safety outcomes. However, those studies were of variable quality and lacked a consistent framework linking CNL care model structures, processes and outcomes. Furthermore, early studies identified variability across key dimensions of CNL structures, practices and anticipated outcomes that created barriers to knowledge synthesis across studies and that threatened the validity of inferences regarding CNL care model effect. The lack of a consistent conceptual framework to guide effectiveness research on CNL integrated care delivery was the impetus for this programme of research. The research described in this paper leverages sophisticated methodologies to fill these gaps in implementation and effectiveness knowledge in ways that can be successfully taken up into real‐world healthcare settings to achieve better care and better health.

### 2.2 Comparing the CNL care model with traditional nursing care models

In the hospital, what most people think of when they think of an RN is a ‘staff nurse.’ This is the RN who is assigned to a patient and is responsible for their care during their ‘shift’, which is typically either 8 or 12 h. This is the standard nursing care delivery model in many hospitals, is called primary care nursing, and is operationalized as nurse‐to‐patient ratios based on patient acuity (i.e. how sick the patient is). The model emphasizes the count of RNs that are required for a specific number of patients on a hospital unit per shift. For hospitals using 12‐h shifts, RNs typically work three shifts per week. Hence, a patient is cared for by multiple staff RNs during the course of their hospitalization. RNs use their competencies to organize the care for each individual patient. The primary care model has been studied in numerous observational studies (see, e.g., Aiken et al., 2002, Needleman et al., 2011). While the evidence is crystal clear that the presence of staff RNs reduces patient mortality/morbidity, there is no evidence that any specific ratio is more effective than another (Needleman & Shekelle, 2019; Shekelle, 2013), suggesting that other factors are likely at play in the complex healthcare environment with respect to effective organization of nursing knowledge and practice.

A CNL is an RN who receives a Master's degree in an accredited CNL nursing programme, allowing them to sit for and pass the national CNL certification exam, administered by the Commission for Nurse Certification. CNLs, in addition to retaining their RN competencies, graduate with three overarching ‘expert‐level’ competencies: clinical leadership, clinical outcomes management and care environment management (Bender, L'Ecuyer, & Williams, 2019). Certification must be renewed every 5 years to continue to use the CNL designation.

The CNL care model is a different way of organizing nursing care. Instead of focusing on RN ratios, the CNL care model integrates CNLs at the unit level to lead the organization of patient care, leveraging staff RN and other clinician's particular competencies and strengths, with the goal of providing consistently safe and high‐quality care to patients. In this model, staff RNs continue to work a certain number of 8‐ to 12‐h shifts a week and focus on care for individual patients. CNLs are also structured at the clinical care unit level and typically there are 1–2 CNLs functioning within each unit. They are not responsible for administrative duties. While they can and do care for individual patients, their main responsibilities involve continual assessments of the clinical unit to identify opportunities for clinical care, clinical throughput and clinical outcomes improvement. They use their master's level CNL competencies to create appropriate teams and make these improvements.

### 2.3 Baseline evidence supporting the CNL care model

A systematic literature review of the CNL evidence, conducted at the onset of this research programme's trajectory, highlighted the feasibility and capacity of CNL integrated care delivery to improve patient care quality and safety (Bender, 2014). The types of studies that measured the outcomes of CNL implementation included: natural experiment case studies using pre–post‐measurement with no control (*n* = 11); quasi‐experimental longitudinal, pre–post, repeated measures interrupted time series (ITS) studies with control (*n* = 1) and without (*n* = 2) and cross‐sectional research (*n* = 1). Safety outcomes included Joint Commission Core metrics for adherence to evidence‐based clinical guidelines including: myocardial infarction, congestive heart failure (CHF), pneumonia and influenza immunization, smoking cessation counselling and venous thromboembolism prophylaxis. Other safety outcomes included falls, ventilator‐associated pneumonia, central line infections, length of stay and 30‐day readmission for CHF and pneumonia. Quality outcomes measured included patient satisfaction (through either the Hospital Consumer Assessment of Healthcare Providers and Systems [HCAHPS] or Press Ganey instrument), nursing hours per patient day and procedure cancellations. Staff satisfaction metrics included staff and physician satisfaction survey results, staff retention/turnover and RN work‐related stress. It is important to note that no study measured CNL practice; improved outcomes were a proxy measure for adequate and sufficient CNL practice. The overall evidence was of very low to moderate‐low quality based on the Grading of Recommendations Assessment, Development and Evaluation (GRADE) guidelines, in part because observational studies (in contrast with RCTs) are labelled under these guidelines as low‐quality studies. However, 14 of 15 studies reported quantified improvements in quality, safety and staff satisfaction outcomes, and showed consistency in the variables used, highlighting the feasibility and capacity of the model to improve outcomes, which prompted our programme of research.

### 2.4 Challenges with traditional approaches to effectiveness research

The question of the form of evidence needed to provide credible, generalizable accounts of a care delivery model's effectiveness in generating care quality is not an easy one to answer. As Chambers and Norton (2016) have recently emphasized, the scientific community too often overlooks the complexity of care delivery by assuming that researchers should work to codify efficacy *before* moving in a linear manner to tests of effectiveness and implementation. This approach assumes that efficacy for any intervention can be determined once and for all, independent of its context. However, nursing care delivery models are systems of care with any number of interdependencies, not discreet interventions can that be studied independently of their context.

One way to shed light on the issue is to highlight the distinction between empirical and analytic generalization. Empirical generalization is about whether the *results* from a study can be expected to hold in situations outside the study. Statistical generalization is a form of empirical generalization; an expectation that the effect size of study results will hold for the entire population from which the study sample was derived. Analytic generalization concerns the applicability of conditional relationships found in one study to other studies or settings (Tsang, 2013; Yin, 2013). Conditional relationships are the logical explanations for the dynamics of action for a phenomenon of interest. Analytic generalizations can be considered analogous to the ‘generalizable determinants’ of a complex care delivery system, which conceivably can be operationalized in diverse ways depending on the case or context. This means that generalizable determinants do not necessarily need to be prescriptive actions attached to a statistically significant effect size, but rather robust conceptualizations of what works that have been found to be operationalizable in different instances. These analytic generalizations could be considered the ‘core concepts’ that underpin any activities or practices undertaken as part of enacting complex change, and it is these ‘core’ conceptions that can be expected to be generalizable to other organizations, rather than a statistical outcome or specific protocol (Ovretveit et al., 2011; Parry et al., 2013; Perez Jolles et al., 2019).

Another difficulty related to this programme of research is that of establishing causal specificity and sensitivity about traditionally conceptualized nurse‐sensitive indicators of quality and safety (Afaneh et al., 2021). The complexity of interrelatedness of factors in the health care delivery arena highlights the importance of the suitability of measures of outcome as well as the suitability of the analytical approach to measuring change when attempting to imply direct causality to the interdependencies among practice, context and outcome variables. This programme of research posits outcome indicators that may be upstream variables of practice characteristics that ‘set up’ microsystems to produce better/more consistent practice‐sensitive patient outcomes. For example, our findings to date suggest that CNL practice is associated with reduced clinical variability of traditional of care quality indicators, even if the indicators themselves did not statistically change (see Results section). This means that consistency around an indicator of quality could be considered as meaningful a quality outcome as the indicator itself. However, there are limited analytic procedures to detect such an outcome.

Hence, before this programme of research could even be launched, there was much to do in terms of finding or developing research designs and methodologies with the capacity to examine non‐linear processes, and to select or produce outcomes that would reflect meaningful changes in these processes. This took time (over a decade and counting) and a willingness to reach out to other disciplines and work together to understand, adapt and even develop methods and measures that could accomplish what was required.

## 3 RESEARCH OBJECTIVES AND APPROACH

### 3.1 Specific aims

This paper describes a trajectory of scholarship with the following aims, (a) to clarify CNL practice, (b) to develop and empirically validate a translational model of frontline care delivery that includes CNL practice and (c) to delineate the patterns of and critical outcomes of successful implementation of the CNL care model. Each study of this programme of research has its own particular aims and methods and findings, which are described in more detail in the ‘Research Methods and Timeline’ section.

### 3.2 Embedding implementation science into effectiveness research

Current literature makes clear that the focus of implementation science – implementing interventions effectively in health settings – comprises non‐linear, complex phenomena. There is growing consensus in the implementation science and health services research fields that inquiry into and evaluation of complex healthcare delivery systems must move past traditional binary questions of efficacy and towards a more sophisticated exploration of ‘generalizable determinants of beneficial outcomes’, which include implementation strategies that facilitate adoption and success (Glasgow, Lichtenstein, Marcus, 2003; Hawe, 2015; Raine et al., 2016). Therefore, assuming dynamic complexity becomes critical to advancing the field.

We have addressed this need via innovative adaptations to methodologies that have the capacity to analyse the complexity of nursing practice. These approaches have been informed by both a process and complexity lens, which makes visible the contingencies and interdependencies that give rise to nursing practice and its implementation (see Braithwaite et al., 2018 for the link between implementation and complexity science). These lenses and approaches have enabled us to show how CNL practice manifests as a relational process and how these processes influence what happens in, and to, the contexts where nurses practice, and how that makes a difference to patient care quality and safety.

### 3.3 Using a system‐based participatory framework to conduct the research

Participatory research is explicit about involving the ‘end users’ of any research in the very development of that research in terms of questions, research aims, and methods and outcomes of interest. Participatory research can involve individuals, communities, and/or systems. Principles to consider when the ‘end users’ are health systems include engaging the health systems at all phases of research, building on resources and goals already present within the health systems, creating and investing in long term and robust partnerships and engaging the research as a cyclical, iterative process (Schmittdiel et al, 2010).

To ensure these principles were in play throughout the research process, our programme of research is scaffolded by a stakeholder‐engaged framework adapted from a 2010 Institute of Medicine (IOM) report outlining approaches towards redesigning clinical research with practice in mind in the context of health systems (Olsen & McGinnis, 2010). See Table 1 for details.

**TABLE 1:** The CNL stakeholder‐engaged framework for research.

**Participatory Framework:** Clinical Nurse Leader Research Collaborative Representation from CNL policy, practice, education, health systems, research	**Goal:** Synthesize existing knowledge	**Aim:** CNL Model Practice and implementation Dynamics of action
		**Aim:** CNL metrics Identify existing variables Develop new variables
	**Goal:** Develop pragmatic research strategy	**Aim:** Appropriate research designs Harness existing date Generate new data
		**Aim:** Appropriate evidence Implementation evidence Effectiveness evidence
	**Goal:** Conduct national‐level research	**Aim:** Generalizable evidence Nationwide research laboratory Combine resources for data collection and analyses Ensure comparable outcomes

Through attendance at the AACN annual CNL summit, academic, clinical and research scholars as well as health system leaders met each other and realized they had a common interest in wanting to know how best to implement CNL practice (Bender, Baker, et al., 2019). We, thus, began concerted efforts to work together on building the CNL evidence base. The group formalized into the CNL Research Collaborative (CNLRC), an AHRQ affiliate practice‐based research network (https://www.ahrq.gov/ncepcr/communities/pbrn/registry/clinical‐nurse‐leader‐research‐collaborative.html). The CNLRC board members represent regional and national health systems across the nation, universities with CNL educational programmes or CNL research hubs and the AACN. The CNLRC is using participatory approaches to capture and leverage experiences and expertise from diverse practice, education, health system, policy and research perspectives at all phases of inquiry.

## 4 SAMPLES AND DATA SOURCES

### 4.1 Funding

The research has been funded through a number of grant mechanisms over its course. Early research was funded by small internal grants provided by the University of San Diego and the University of California, Irvine. Later research was funded through three grants provided by the Commission on Nurse Certification (2014–2015, 2016–2017, 2018–2024). The most recent research has been funded through an R01 grant (PI, Bender) by the Agency for Healthcare Research & Quality (2020–2024).

### 4.2 Institutional review board approvals

All research activities have been reviewed by appropriate Institutional Review Board entities and approved as exempt research.

### 4.3 Samples and settings

The sample for the earlier stages of this programme of research (which we call the 2015 dataset) included the known population of certified CNLs in the United States. CNLs are certified by the accredited Commission on Nurse Certification (CNC) to ensure national‐level standards for CNL competencies, as delineated in the AACN Competencies and Curricular Expectations for Clinical Nurse Leader Education and Practice (AACN, 2013). The CNC manages the certified CNL database, which included a population of 3375 CNLs at the time they were studied. The samples also included health system clinicians and administrators across the United States that self‐identify as being involved in a CNL initiative at the time they were studied (for details see Bender, 2016a, 2016b; Bender et al., 2017).

The sample for the more recent stages of this programme of research (which we call the AHRQ dataset) was recruited from a purposive set of clinical care units in United States hospitals that were redesigning their unit‐level RN staffing model to integrate CNLs with workflows aligned with the CNL care model. The study settings were selected for representation of geographic region and setting ownership status diversity (for details, see Bender et al., 2021).

## 5 RESEARCH METHODS AND TIMELINE

**FIGURE 1:**
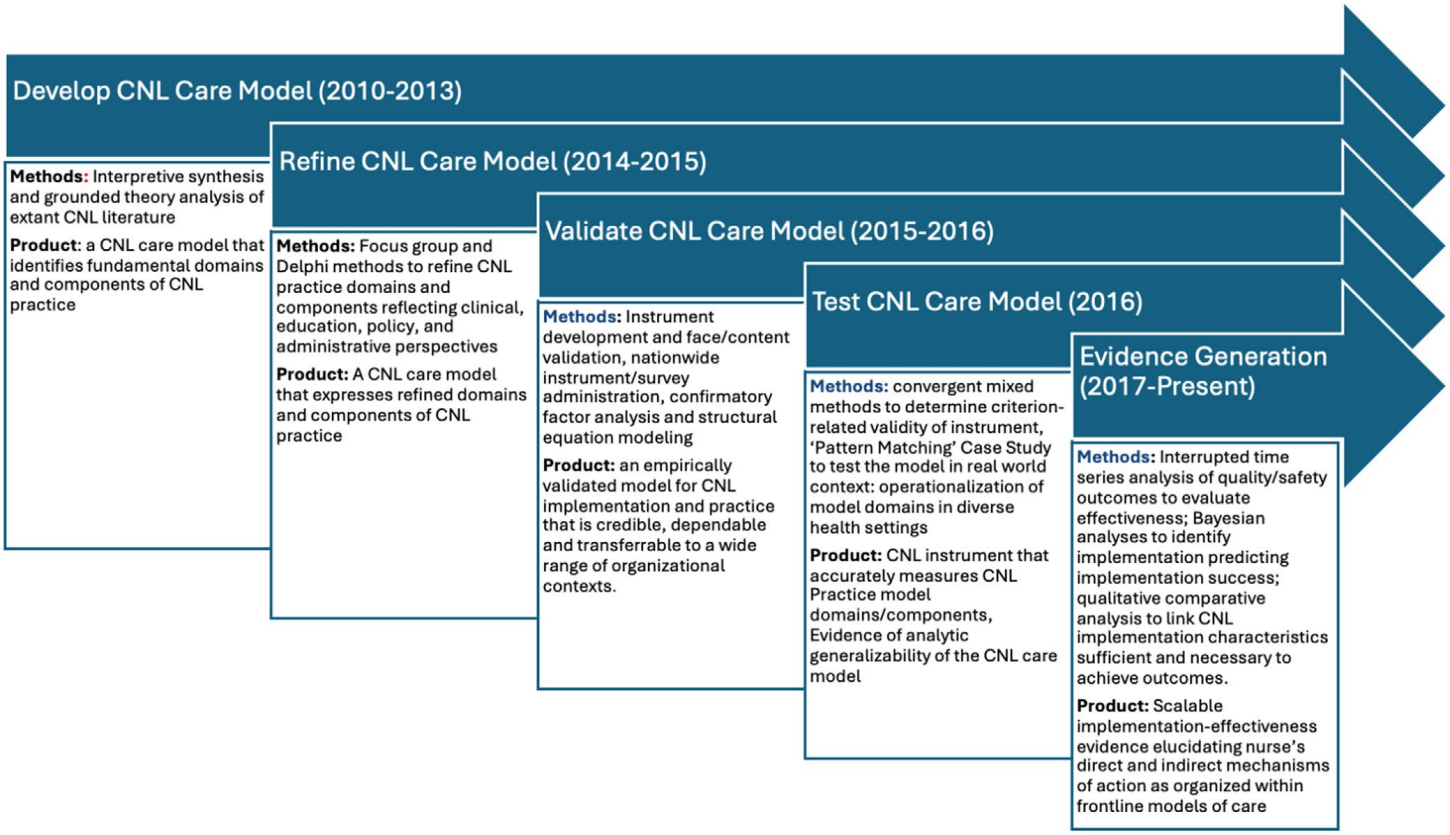
Methodological timeline to achieve research programme aims.

To accomplish aims 1 (clarify CNL practices) and 2 (develop and validate a CNL care model) of this programme of research, we conducted four studies. To accomplish aim 3 (delineate the patterns of and critical outcomes of successful implementation of the CNL care model) of this programme of research, after developing appropriate study designs and methodologies, we are currently conducting a national‐level implementation–effectiveness study. The timeline for these studies can be seen in Figure 1, and the methods for each study are described in the following subsections.

### 5.1 Developing and refining the CNL care model

The CNL care model was developed to fully understand CNL implementation and was developed through a sequential mixed methods approach that included an interpretive synthesis design employing grounded theory methodology on existing CNL research and the extensive grey literature to produce a preliminary CNL care model (for details, see Bender, 2016a, 2016b), followed by an expert panel approach to refine the CNL care model (for details see Bender et al., 2017).

### 5.2 Validating the CNL care model and survey instrument

Subsequent to refinement of the CNL care model, it was transformed into a 70‐item survey instrument using a modified Delphi approach (for details, see Bender et al., 2018) and validated through a national‐level mixed methods study (for details, see Bender et al., 2018). Structural equation modelling of the data was used to confirm the directionality and significance of all hypothesized model pathways that establish the dynamics of action explaining how the CNL care model, appropriately structured and implemented, led to expected outcomes. Confirmatory factor analysis was used to validate the survey items as expressing the latent concepts of the CNL care model.

### 5.3 CNL care model testing

Once the CNL care model and survey instrument were validated, we conducted a study to test their correspondence to a real‐world case consisting of one regional United States health system that integrated clinical nurse leaders into their care delivery system in 2010 (for details, see Bender et al., 2018). We used a pattern‐matching case study approach because it is well suited for research aimed at generating advanced understanding of empirical manifestation of conceptual constructs, such as the CNL care model domains, especially the potentially diverse manifestations depending on local context‐dependent implementation dynamics (Barratt et al., 2011). Case study does not explicitly control or manipulate variables. It does, however, study a phenomenon in its natural context, at one or a few sites, and can make use of qualitative and quantitative methods for data collection (Cavaye, 1996). Even without explicit manipulation of variables, it provides a powerful approach to determining the explanatory power of a model of a complex phenomenon, such as the CNL care model, and therefore, provides insights into its overall generalizability (Bitektine, 2007).

#### 5.3.1 Type 2 implementation–effectiveness research approach

With the survey tools in place to conduct research on the CNL care model, we are now using a hybrid implementation–effectiveness research approach to generate the national‐level evidence base for the model and to specify effective implementation strategies that health systems can match to their specific characteristics, including strategies that minimize resource use while still achieving intended outcomes. The hybrid implementation–effectiveness research design was formalized in a 2012 paper published in *Implementation Science* (Curran et al., 2012), and combines into a single research programme questions that concern the effectiveness of an intervention as well as how to best implement it. The goal is to reduce the time span from promising innovation to routine practice. The hybrid approach can prioritize effectiveness or implementation, depending on aspects such as the level of evidence for a specific intervention, the extent to which it is expected to be adapted depending on contexts and the extent to which existing implementation determinants are known (e.g. facilitators and barriers).

For this final stage of the research programme, a type 2 hybrid approach is being used because while there is already a small evidence base associating the CNL care model with improving care quality outcomes (Bender, 2014), the CNL care model makes clear that how the model is implemented directly influences the outcomes achievable and thus is as important to track as effectiveness. This meant that there needed to be an equal focus on both implementation and effectiveness. With this approach, we were able to focus on what works, where and why, all together, rather than assume that efficacy must come before effectiveness (Curran et al., 2022 and for more study details, see Bender et al., 2021).

#### 5.3.2 Interrupted time series analysis to measure CNL care model effects

To determine the effectiveness of the CNL care model, we could not rely on traditional approaches such as a randomized clinical trial using a ‘manualized intervention’. This is because it is known that the implementation of the model, and thus the very intervention itself, varies based on pre‐existing health system contextual characteristics, and this variability directly influences subsequent CNL practices and achievement of outcomes (Bender, 2014).

Furthermore, clinical practice is a dynamic process that is temporally driven. This temporality creates problems in terms of statistical analysis, as many traditional analytic techniques have an assumption of independent observations. This assumption is violated with clinical processes that cannot be separated easily into independent quanta of care and outcome. This dependence of measures over time is called autocorrelation. Interrupted time series research design accounts for autocorrelation in analysis and is well suited for time‐dependent evaluations of clinical process interventions (Biglan et al., 2000). In interrupted time series (ITS) analyses, the outcome variable is manipulated (via introduction of the intervention) after a series of baseline data measurements. Data measurement then continues through similar time increments after introduction of the intervention. The ITS design cannot be used to detect a cause–effect relationship between variables, but it can be determined that an intervention is empirically correlated with changes in the outcome. The design improves the internal validity of non‐randomized study methodology by accounting for potential study biases, such as pre‐intervention trends, seasonality and random fluctuation, through the collection and analysis of data over time.

However, standardized methods for analysing ITS data do not model changes in variation and correlation following the intervention. This is a key limitation since it is plausible for data variability and dependency to change because of the intervention. Moreover, Traditional ITS methodology either assumes a pre‐specified interruption time point with an instantaneous effect or removes data for which the effect of intervention is not fully realized. Therefore, we developed a novel robust interrupted time series (robust‐ITS) model that overcomes these omissions and limitations. The robust‐ITS model formally performs inference on (1) identifying the change point; (2) differences in pre‐intervention and post‐intervention correlation; (3) differences in the outcome variance pre‐intervention and post‐intervention and (4) differences in the mean pre‐intervention and post‐intervention (Cruz et al., 2017).

The novel Robust‐ITS statistical modelling approach has enabled the creation and analysis of an, until‐now untested, outcome variable; change in variability around an outcome of interest before (up to 24 months) and after (up to 24 months) implementation of the CNL care model. Standard approaches to ITS segmented regression assume a pre‐specified interruption time point or censor data for which the effects of the intervention are hypothesized not to be fully realized and assume no changes in variance pre–post‐intervention. Models incorporating these assumptions can bias effect estimates. Our approach is an advance over standard ITS segmented regression approaches because it explicitly models the lag between an intervention's introduction and its effect in practice, as well as changes in variation and correlation pre‐ and post‐intervention.

#### 5.3.3 Bayesian analysis to predict implementation patterns correlating with implementation success

For the validated CNL survey instrument, in addition to items corresponding to model domains and components, one survey item asks respondents to rate how successful the CNL initiative was in their facility on a scale of 0–100 using a slider bar. The survey data are complex, with three nested hierarchical levels. Furthermore, patterns of response were not assumed to follow a Gaussian distribution, which precludes the use of standard analytic procedures.

Because of this complexity, we modelled the hierarchical structure of the data using a Bayesian multilevel regression mixed modelling approach (for details, see Williams et al., 2023). A zero–one‐inflated beta distribution, a mixture of Bernoulli distributions for the minimum and maximum responses and a beta distribution for the responses between the minimum and maximum, was used to fit success ratings in the model.

Beta models allow modelling a proportion that is between, but not including, 0 and 1. Treating item scores as proportions is often more appropriate than treating them as coming from a Gaussian distribution, because they are bounded by minimum and maximum scores on the item scale. Since a beta distribution cannot model proportions that are exactly 0 or 1, these are modelled as a separate Bernoulli process using two additional parameters. These parameters model the probability that a zero or a one occurs, and out of a zero or one, the probability that one occurs. This mixture of distributions is especially important if there are many maximum and minimum ratings. Item and success scores were converted to proportions by dividing by their maximum value. In these data, items are nested within components which are nested within domains (a domain/component/Item hierarchical structure). Varying intercepts and varying slopes for the effect of success score were included for the nested effects. Varying intercepts were included for each participant as well. The standard deviations for the varying intercepts and slopes at each level are reported in the results. These standard deviations represent the estimated variability in the outcome among a population of members at that level. For example, the estimated standard deviation of the varying intercept at the domain level represents the estimated variability in item rating among different domains when the success score is zero (i.e. baseline item rating). Similarly, the estimated standard deviation of the varying intercept at the component level represents the estimated variability in item rating among different components within the same domain, when success score is zero.

#### 5.3.4 Qualitative comparative analysis to link implementation dynamics with effects

Qualitative comparative analysis (QCA) offers an analytic approach for detecting implementation patterns that may be invisible to conventional statistical techniques such as linear regression (Rubinson & Ragin, 2007). QCA's value for our programme of research is that it can be used to analyse complex implementation relationships across a sample of heterogeneous cases, such as clinical units nested within hospitals. QCA is based on Boolean, rather than linear algebra and operates by identifying and measuring the strength of subset relationships. These subset relationships may be interpreted in terms of necessity and sufficiency.

Our hypothesized primary relationship is that clinical units with improved quality and safety are a perfect subset of clinical units with adequate CNL practice (i.e. continuous clinical leadership; see Table 2). This means that CNL practice is a necessary condition for outcomes improvement. The other main hypothesized relationship is that clinical units with certain configurations of CNL implementation will produce adequate CNL practice, while others will not. This is critical knowledge: if we determine that, on average, there are not adequate levels of CNL practice in clinical units, the QCA analysis will allow us to determine if the lack of outcomes is due to the ineffectiveness of CNL practice or is related to inconsistencies in or sub‐thresholds of CNL practice, or perhaps contextual barriers prohibiting the implementation of CNL practice.

**TABLE 2:** Validated CNL care model domains and components.

CNL care model domain	CNL care model component
Readiness	Understanding care delivery gaps
	Consensus CNL practice can close gaps
	Organization‐level implementation strategy
Structuring	Microsystem‐level structures
	CNL competency structuring
	CNL workflow structuring
CNL Practices: Continuous clinical leadership	Facilitate effective ongoing communication
	Strengthen intra‐ and interprofessional relationships
	Create and sustain teams
	Support staff engagement
Outcomes	Improved care environments
	Improved care quality and safety
Value	CNL is perceived by clinicians and administrators as adding value to the care that is delivered

## 6 FINDINGS

### 6.1 Aim 1 and aim 2

The early stages of research produced a validated CNL care model (see Table 2). The interpretive synthesis produced a preliminary conceptual model that was then refined through an expert panel process to produce a preliminary CNL care survey with items corresponding to the refined model, which incorporates five domains and 13 sub‐domains into a temporal pathway that demonstrates how the CNL care model, appropriately structured and implemented, leads to expected outcomes.

The model's domains are ‘Readiness for CNL integrated care delivery’; ‘Structuring CNL integrated care delivery’; ‘CNL Practice: Continuous Clinical Leadership’; ‘Outcomes of CNL integrated care delivery’ and ‘Value’ (see Table 2). The survey derived from the model was tested with a sample of 921 participants of CNLs, clinicians, and administrators who self‐identified as being involved in a CNL initiative at the time of survey administration. Those who did not self‐identify were exited from the survey (*n* = 299, for details, see Bender et al., 2017). Sample data had good fit with specified model and two‐level measurement structure. All hypothesized pathways were significant, with strong coefficients suggesting good fit between theorized and observed path relationships.

The test of the CNL care model was done through pattern matching case study design and mixed methods in one regional United States health system that integrated clinical nurse leaders into their care delivery system (for details, see Bender et al., 2018). The final CNL survey sample included 209 participants and a total of 57 people directly involved with CNL model implementation were interviewed. The findings confirmed the empirical presence of all clinical nurse leader care model constructs and provided rich descriptions of how the health system implemented the constructs in practice. The findings support the hypothesized model pathway from CNL readiness and structuring to CNL practice and outcomes.

### 6.2 Aim 3

The psychometrically validated CNL survey was used in the implementation–effectiveness study involving 66 clinical hospital units in nine hospitals representing five regional health systems in five states (Texas, Georgia, Michigan, Illinois and South Carolina). We obtained 1186 valid survey responses from clinicians and administrators who answered ‘yes’ to being involved with their organization's CNL implementation at the time of adoption. We also conducted 399 interviews, ranging from brief 10‐min chats answering a single question to extensive 2‐h focus group interviews with respondents from multiple disciplines, all who were working at the time of their organization's respective CNL implementation period, and provided their perspectives of CNL implementation in terms of what went well and what did not go so well with the CNL care model's adoption into practice.

#### 6.2.1 Effectiveness of the CNL care model

An ITS analysis of seven nursing‐specific patient satisfaction metrics (from the Press Ganey survey) in 10 clinical care units of a single hospital that redesigned their nursing frontline delivery to integrate CNLs found that the lowest performing units showed significant increases in quality scores, but there were no significant increases at the hospital level (Bender, Murphy, et al., 2019). The ITS analysis also showed that quality metric of consistency‐in‐outcome increased significantly for every indicator at the hospital and unit level. Putting it another way, variability around the outcome decreased after implementation of the model, which suggests that the CNL care model is associated with the production of stable clinical unit‐level practices that help to reduce clinical variability, as the path to increasing care quality.

#### 6.2.2 Predictors of CNL care model implementation success

The 2015 survey dataset was modelled using a Bayesian multilevel regression mixed modelling approach (Williams et al., 2023). The Bayesian analysis determined the CNL care model elements that best discriminated between lower and higher implementation success. Since Bayesian methods provide posterior probability distributions for each parameter in the model, it was possible to propagate the uncertainty through the calculation of effects for each level of the data (CNL care model domains, components and items), which enabled us to provide uncertainty intervals for all estimates and thus determine which level of modelling provided the best discrimination. The variability around success score across CNL care model element ratings was greatest at the component level, 0.29 (0.18–0.48), compared to either the domain level, 0.16 (0.01–0.54), or the item level, 0.09 (0.01–0.17). The components most predictive of implementation success were (a) consensus CNL model can close gaps, (b) organization‐level implementation strategy and (c) alignment of empirical CNL microsystem level structuring to the model's conceptualization.

The AHRQ survey dataset was also modelled using the same Bayesian approach (Bender, Williams et al., 2024). Interestingly, we found different patterns of implementation correlated with success. The variation (standard deviation) around success score across CNL practice model element ratings was greatest at the domain level, 0.55 (0.17–1.44), compared to either the component level, 0.28 (0.12–0.56), or the item level, 0.31 (0.25–0.39). The outcome domain was most predictive of implementation success. At the item level, elements that showed greatest predictive power to discriminate empirical implementation success within the domains of readiness, structuring and practices included: a shared vision of the model at executive, department and point‐of‐care levels; CNL workflows that are tailored towards specific frontline clinical care delivery needs and CNL practices that empower staff nurses to practice at their full scope of practice.

The noted differences between the two analyses based on different datasets can be explained by the following. The 2015 dataset comprised a nationwide sample of certified CNLs and clinicians/administrators self‐identifying as involved in the implementation of a CNL initiative. More than 80% of this sample identified as a certified CNL (Williams et al., 2023) but only 67% reported practising in a formal CNL role. Furthermore, only 38% of all respondents were reporting on an ‘established’ CNL care model; the majority of respondents (62%) were in the developmental stages of implementation. This is in contrast to the AHRQ dataset, which involved participants of health settings that had actively established the CNL care model and were in the stage of formative and summative evaluations of the model.

With this understanding of the difference across samples for the two data sets, it makes sense that the 2015 survey dataset would manifest different predictors of success than the AHRQ dataset. It is important to realize that the survey participants in 2015 were involved in CNL practice implementation efforts without the benefit of a consistent conceptual framework to guide the efforts. This earlier sample provided robust evidence of the importance of specific components of readiness and structuring that were lacking in many early efforts. The yardstick for success at that time in the history of the CNL initiative was largely focused on getting CNL roles established. The emphasis in the AHRQ data on the importance of practice and outcomes reflects maturation of the initiative with enhanced focus on indicators of the effectiveness of CNL practice integration in the nursing care delivery model. While elements of readiness and structuring remain important as upstream factors, the shift to downstream elements of practice effectiveness is not surprising.

## 7 CONTRIBUTIONS OF THE RESEARCH TO IMPLEMENTATION SCIENCE

In addition to providing robust implementation–effectiveness for the CNL care model specifically, the broader programme of research produced findings that address implementation science directly and also help to better understand the complex inter‐dependent relations between the overarching implementation science concepts of intervention, context and implementation.

### 7.1 The interdependent relationality of implementation science concepts

From the very first papers describing implementation science, it has always been described as involving three fundamental concepts: evidence, context and implementation strategies (Eccles & Mittman, 2006). These overarching concepts are embedded in many implementation science models and frameworks, from original change frameworks (e.g. Pettigrew, 1987) to current models and theories such as iPARISH (Harvey & Kitson, 2015), the normalization process theory (May et al., 2016) and The Consolidated Framework for Implementation Research (Damschroder et al., 2009).

Typically, researchers study these concepts separately, for example, focusing on the contextual barriers to implementation, or strategies that facilitate implementation, or adaptations to interventions. Yet, there is a growing understanding of how these concepts are not separate and independent, but rather mutually defining (e.g. Dixon‐Woods et al., 2011; Greenhalgh et al., 2004; Grol et al., 2007; Hawe et al., 2009).

With this understanding, we examined in depth how one health system changed its nursing model of care, paying attention to the relations between the frontline clinical context, the nursing ‘intervention’, and the implementation processes used (Bender, Burtson, & Lefkowitz, 2019). Using a prospective case study design at a large urban hospital that changed their nursing model to implement the CNL care model on five clinical units, we found that implementation involved system‐level strategies that were well planned and operationalized. However, the CNL care model was not well received initially, even though everyone was aware of, and sometimes even participated in planning its implementation. We found that within the clinical context, it was pre‐existing contextual practices that could not 'take up' what the CNLs were offering that was causing this lack of uptake. But over time, we found a reciprocal process of co‐constituting the CNL ‘intervention,’ which involved slow transformation of the context. Importantly, this process produced something quite different than ‘an implemented intervention.’ The implementation strategies were not the active mechanism driving this process. Instead, the sustained implementation strategies created a condition of opportunity that allowed the intervention to be co‐constituted within its contexts and thus successfully adopted over time. Notably, the same process resulted in heterogenous outcomes, in that the process produced different CNL workflows with varying changes in the frontline clinical context, and it is precisely these interactions which explain the findings. While this certainly ‘muddies’ the boundaries between concepts, this study showed that a focus on interdependent relationality can yield critical knowledge with explanatory power about implementing evidence into practice.

### 7.2 Clinical routines as an under‐explored yet critical component of context

The implementation science literature is clear that ‘context matters’, but a recent scoping review also made clear that ‘there is considerable variation with regard to … how context is defined and conceptualized, and which contextual determinants are accounted for in frameworks used in implementation science’ (Nilsen & Bernhardsson, 2019, p. 18).

With this understanding in mind, we examined the role of context in the implementation of the CNL care model in 11 hospitals across five states (our AHRQ dataset). Through an analysis of interview data and observational field notes, we found that one of the most consistent contextual components influencing implementation across all settings was the clinical routine (Bender & Lefkowitz, 2020). What was of interest was that we could not find a definition of ‘clinical routine’ in a search of both PubMed and the journal *Implementation Science*, beyond three articles in which a routine was considered an individual clinical behaviour. However, the clinical routines we found were not individual behaviours. We identified an appropriate definition of clinical routines in the field of organization science, ‘an organizational routine is a repetitive, recognizable pattern of interdependent actions, involving multiple actors’ (Feldman & Pentland, 2003, p. 105).

Clinical routines we found included interdisciplinary rounding, admissions and discharges, handoffs and medication administration. CNL practices may or may not have ‘touched’ these pre‐existing routines as the nursing model was changed to implement them. If CNL practice did touch these routines, we found that the routines ‘pushed back’. This means that effective pre‐existing routines were prioritized over CNL practices, which could be implemented only to the extent that these pre‐existing routines could stay effective.

One example of this was attending‐resident educational rounds. Resident physicians would routinely review charts on clinical units in preparation for the rounding routine. They found CNLs doing the same thing in the mornings and so their practices changed to be able to routinely chat with each other and share information. This led to the enhancement of other existing routines, notably the discharge routine. Because of this information sharing and later efforts done by the resident and CNL related to discharges, completion of items needed before a patient could be discharged became more efficient and discharges began happening sooner. This was celebrated by all, physicians, nurses and administrators. However, we found that this enhanced discharge routine shifted the timing of other existing routines antecedently, that is, the attending‐resident rounding, which occurred later in the morning related to the residents' sharing information with CNLs on the units to prepare for this rounding (and help facilitate discharge). And while the attending physicians did not mind the delay, it affected their downstream routines (surgery, consultations, etc.).

The overall outcome of all this was that the enhanced discharge routine was stopped because the pre‐existing attending‐resident routine became less effective. This demonstrates how much context matters and suggests a complex causality between interventions and contexts that manifests via unanticipated intersections among existing multiprofessional clinical routines. Importantly for implementation science, clinical routines are not listed as a component in existing context determinant frameworks. This suggests further investigation and conceptualization is needed to advance knowledge about the causal significance of clinical routines for healthcare interventions in implementation science.

### 7.3 Trust as implementation strategy

Trusting relations have been recently identified as important to the operationalization of implementation strategies yet are undertheorized in implementation science (Metz et al., 2022). For example, while the 2022 updated CFIR lists constructs involving shared values, beliefs and high‐quality networks/teams, trust is not a construct. Perhaps because of this, little implementation research examines trust.

Therefore, in a recent analysis, we aimed to characterize trust in terms of what it consists of, how it manifests and how it relates to implementation processes (Bender, Jubb, Rasoulie, Meyers, 2023). In the AHRQ dataset, trust was mentioned frequently by deliverers and receivers as critical to innovation implementation. As one deliverer put it, ‘You've got to establish everybody's trust. You cannot have [implementation success] without trust’. We identified temporal dimensions of trust building, achievement and sustainment. The first dimension involves receptivity; deliverers demonstrating the innovation in ways that show authentic concern for recipient perspectives and needs. Frequently this involved demonstrations of how the innovation could save receivers time and effort. The second dimension involves activity; receivers taking up elements of the innovation when their workflow allowed. Frequently this involved clinical circumstances when receivers experimented with the innovation to help resolve issues. The third dimension involves interactivity: voluntary deliverer/receiver collaboration to adopt the innovation into routine clinical practices. This dimensionality was not linear and not manifested across all units. Findings suggest that trust can be built over time and manifests through stages of receptivity, activity and interactivity. Findings help to conceive trust as a staged process or implementation strategy that can be facilitated and even measured as a marker towards implementation success. This operationalization of trust aligns with recent conceptual implementation scholarship on trust building and provides empirical evidence for an important emerging concept in implementation science.

## 8 CONCLUSION

In this paper, we have provided a detailed account of the multi‐level, multi‐faceted and temporal nature of the implementation of a nursing care model, as well as the multi‐level, multi‐faceted and temporally sensitive research approaches and methodologies that were needed to generate actionable evidence about how to effectively implement such a care model, or not, and the resulting quality and safety outcomes that accrue.

As a final consideration, we believe that our programme of research provides an important exemplar in terms of bringing nursing practice in and of itself back to the forefront of nursing scholarship. As Gortner makes clear in a historical paper on nursing research in the United States, with the launch of the National Institute of Nursing Research (NINR) came a distinct change in foci for nursing research, from nursing practices towards ‘the nature of the phenomena we wish to influence’, such as heart transplantation and aggressive chemotherapy regimens (Gortner, 2000, p. 63). This has led to important and life‐saving knowledge on clinical treatments and protocols developed with a nursing lens that make a difference to patient care and outcomes.

However, this orientation, dominant since 1986, has occurred during unprecedented changes in the way healthcare is organized and funded and the ways clinician workflows within those changed environments have been reorganized, eliminated, or introduced. A continued bias against research on nursing practice itself, called as recently as 2020 by one nursing journal editor as ‘navel‐gazing’ (Pickler, 2020, p. 7), needs to be overcome. To help with this, we are heartened to see the NINR is once again prioritizing research on care models that leverage ‘the on‐the‐ground experiences of nurses’ (https://www.ninr.nih.gov). Our research demonstrates how frontline nursing models of care leverage nurses and nursing knowledge in ways that can achieve health and wellness through nursing‐inflected structures and processes.

## AUTHOR CONTRIBUTIONS

All authors were involved in the development and writing of the manuscript.

## FUNDING INFORMATION

This programme of research received funding from the Agency for Healthcare Research and Quality: 1 R01 HS027181‐01A1 PI: Bender, with a project period from 9/30/2020 to 9/29/2024. The funding body was not involved in the design of the research nor collection, analysis, interpretation of data or writing of the manuscript.

## CONFLICT OF INTEREST STATEMENT

All authors declare that they have no conflict of interest.

## PEER REVIEW

The peer review history for this article is available at https://www.webofscience.com/api/gateway/wos/peer‐review/10.1111/jan.16395.

## ETHICS STATEMENT

All research activities have been reviewed by appropriate IRB entities and approved as exempt research.

## Data Availability

Raw data generated to support the findings of this study will be available from the corresponding author [MB] on request.
